# Construction and Validation of the Touch Experiences and Attitudes Questionnaire (TEAQ): A Self-report Measure to Determine Attitudes Toward and Experiences of Positive Touch

**DOI:** 10.1007/s10919-018-0281-8

**Published:** 2018-08-01

**Authors:** P. D. Trotter, F. McGlone, R. L. E. P. Reniers, J. F. W. Deakin

**Affiliations:** 1 Department of Natural Sciences and Psychology, Liverpool John Moores University, Byrom Street, Liverpool, UK; 2 Department of Psychology, Manchester Metropolitan University, Manchester, UK; 3 Institute of Psychology, Health and Society, University of Liverpool, Liverpool, UK; 4 Institute of Clinical Sciences, University of Birmingham, Birmingham, UK; 5 Institute for Mental Health, University of Birmingham, Birmingham, UK; 6 Neuroscience and Psychiatry Unit, The University of Manchester, Manchester, UK

**Keywords:** C-tactile afferents (CTs), Social touch, Interpersonal touch, Affective touch, Childhood trauma, Social support

## Abstract

Despite growing interest in the beneficial effects of positive touch experiences throughout our lives, and individual differences in how these experiences are perceived, there is not yet available a contemporary self-report measure of touch experiences and attitudes, for which the factor structure has been validated. This article describes four studies carried out during the construction and validation of the Touch Experiences and Attitudes Questionnaire (TEAQ). The original TEAQ, containing 117 items relating to positive touch experiences was systematically constructed. Principal component analysis reduced this measure to 57 items and identified six components relating to touch experiences during childhood and adult experiences relating to current intimate touch and touch with friends and family. Three attitudinal components were identified relating to attitude to intimate touch, touch with unfamiliar people, and self-care. The structure of this questionnaire was confirmed through confirmatory factor analysis carried out on data obtained from a second sample. Good concurrent and predictive validity of the TEAQ compared to other physical touch measures currently available was identified. Known-group validity in terms of gender, marital status and age was determined, with expected group differences identified. This study demonstrates the TEAQ to have good face validity, internal consistency, construct validity in terms of discriminant validity, known-group validity and convergent validity, and criterion-related validity in terms of predictive validity and concurrent validity. We anticipate this questionnaire will be a valuable tool for the field of physical touch research.

**Electronic supplementary material:**

The online version of this article (10.1007/s10919-018-0281-8) contains supplementary material, which is available to authorized users.

## Introduction

Since the discovery C-tactile afferents (CTs) in humans by Nordin (1990), there has been growing interest in the role of positive touch experiences throughout our lives. These nerve fibres respond optimally to the sensations experienced during a human caress; gentle stroking touch delivered at the temperature of human skin (Ackerley et al. 2014; Loken et al. 2009; Vallbo et al. 1993, 1999). Additionally, CTs are slowly conducting and their stimulation leads to activation of limbic-related brain regions, rather than the somatosensory cortex (Bjornsdotter et al. 2009; McGlone et al. 2014; Olausson et al. 2002, 2008). Based on this evidence, CTs have been proposed to have a key role in encoding the rewarding properties of positive social touch (Morrison et al. 2010).

The importance of positive tactile experiences during early development was recognised by Spitz (1945) who reported children in orphanages who had their basic needs met, but received little nurturing touch, failed to thrive. Harlow (1958) highlighted the importance of contact comfort during early development, originally observing the strong attachment neonatal monkeys developed to the cloth pads in their cages. Inanimate ‘surrogate mothers’ were then developed, to which neonatal monkeys became attached and could be pacified by these ‘mothers’ when stressed, but only when these mothers were covered in a soft layer of cotton terry cloth, with no real benefit of a wire ‘mother’ at all (Harlow 1958; Harlow and Suomi 1970). Interestingly, manipulation of the temperature of these ‘mothers’ also altered the response of the monkeys to their ‘mother’, with a warm mother (~ 24 °C) promoting attachment and a cool mother [~ 17 °C, sub-optimal for CT activation (Ackerley et al. 2014)] inducing a stress response and no attachment (Harlow and Suomi 1970).

The work of Meaney (for a review, see Meaney and Szyf 2005) has provided further evidence of the importance of positive touch experiences in early development. Rat pups experiencing high levels of maternal licking and grooming touch in the early neonatal period have significantly reduced stress responses in adulthood compared to rat pups receiving low levels of licking and grooming touch, due to the induction of an epigenetic process (Meaney and Szyf 2005). This protective effect of maternal stroking, in terms of both the epigenetic and behavioral effects, has now been replicated in humans (Murgatroyd et al. 2015; Sharp et al. 2012).

In addition to this protective effect of positive touch in early development, a protective effect in adulthood has also been identified. Cochrane (1990) identified that adults experiencing unsatisfactory levels of social touch either during childhood or at present, had greatly increased vulnerability to depression. In terms of the therapeutic value of positive touch, massage therapy reduces salivary cortisol, increases urinary serotonin metabolite levels, and reduces depression, stress, anxiety, aggression, and pain (Diego et al. 2002; Field et al. 1996, 2004, 2005; Hernandez-Reif et al. 1998, 2001). Eaton et al. (1986) identified elderly care home residents consumed more calories and protein if they were touched on the shoulder before eating.

Evidence of a role of serotonin, oxytocin, and endogenous opioids in CT activating touch responses (Nummenmaa et al. 2016; Trotter et al. 2016; Walker et al. 2017), provides a potential mechanism through which positive touch produces the beneficial effects identified above. Oxytocin promotes social bonding, produces feelings of well-being, has an anxiolytic effect at low doses, a sedative effect at high doses, is a natural analgesic, and reduces arousal through promoting parasympathetic activity (Uvnäs-Moberg et al. 2015). Many of these effects are also produced by CT optimal stroking touch (Fairhurst et al. 2014; Liljencrantz et al. 2017; Pawling et al. 2017a, b), further implicating oxytocinergic mechanisms, as well as highlighting the beneficial effects of positive touch experiences.

Despite the growing evidence of the importance of positive touch experience throughout our lives, to date there are no contemporary positive touch questionnaire measures relating to attitudes to and experiences of positive touch for which the factor structure is known. Additionally, none of the currently used measures have been extensively validated. This study describes the construction and validation of the Touch Experiences and Attitudes Questionnaire (TEAQ), designed to measure positive touch experiences both at present and during childhood, as well as obtain measures of attitudes towards positive touch. Most questions refer to interpersonal touch, but Harlow’s work has highlighted inanimate positive touch experiences can also be beneficial (Harlow 1958; Harlow and Suomi 1970). We know CTs are activated by robotic touch (Ackerley et al. 2014; Loken et al. 2009), therefore interpersonal touch is not necessarily key to inducing the beneficial effects of positive touch. For this reason, questions surrounding non-interpersonal positive touch were also included in the original questionnaire measure.

It should be noted that we have little knowledge at present of how CTs respond to static touch compared to stroking touch, although it is known that static touch does activate these nerve fibres (Bessou et al. 1971; Wiklund Fernström et al. 2002). Additionally, we have no evidence at present that these nerve fibres respond more to low force compared to high force touch, simply that these nerve fibres are able to respond to low forces that do not activate C-nociceptors (Vallbo et al. 1993, 1999). For these reasons, this questionnaire measure is focused on all positive touch experiences, rather than having a specific focus on gentle stroking touch sensations. This not only improves the ecological validity of this measure and allows this measure to be used by researchers in a variety of fields relating to positive touch experiences, but also has the capacity to help us understand the relative importance of a range of positive touch behaviors and experiences during our lives.

This article describes four studies carried out during the construction and validation of the TEAQ. During the first study the original TEAQ, containing 117 items relating to positive touch experiences was constructed. Principal component analysis reduced this measure to 57 items and identified six components relating to touch experiences during childhood and adulthood and attitudes towards current positive touch. The structure of this questionnaire was confirmed in Study 2 through confirmatory factor analysis carried out on data obtained from a second sample. Study 3 describes further validation of the TEAQ, determining the concurrent and predictive validity of this measure compared to other physical touch measures currently available. Finally, Study 4 examines gender, age and marital status differences in TEAQ responses.

## Study 1

### Introduction

The aim of this study was to identify all circumstances in which positive touch is experienced. From this, items were written based on these circumstances. Following data collection, principal component analysis was used to determine the component structure of the questionnaire and remove any superfluous items.

### Method

#### Participants

The 117-item draft TEAQ was completed by 867 participants. Of these participants, 249 had > 5% of their data missing and were excluded. All participants included in the analysis had less than 1.2% missing data. For the whole dataset, only 0.3% of data was missing once the 249 participants had been excluded.

Of the 618 participant responses included in this study, 440 were female and 178 were male. Mean age ± standard deviation was 26.9 ± 9.3 years. Age, sex, and socio-economic demographics of the sample are presented in Table 1. The majority of participants were either students (58%), or in full-time employment (30%). Most participants were in a relationship (60%, including 26% married/cohabitating), 37% were single, 2% were separated/divorced, and 0.2% widowed. Additionally, the majority of participants had no children (82%).

**Table 1:** Participant demographics for each of the four studies

	Study 1 sample	Study 2 sample	Study 3 sample	Study 4 sample
Sample size	618	704	201	1509
Mean age ± SD	26.9 ± 9.3	27.4 ± 9.6	25.5 ± 10.1	27.0 ± 9.6
Gender (%)				
Male	28.8	26.3	19.9	26.6
Female	71.2	73.7	79.6	73.2
Employment status (%)				
Working F/T	39.0	25.0	16.4	28.4
Working P/T	5.8	6.5	5.0	6.3
Student	52.4	62.4	78.1	61.0
Not working	2.7	5.8	0.5	4.2
Marital status (%)				
Single	39.0	36.1	39.8	37.6
In a relationship	32.8	35.2	40.3	35.0
Married/cohabiting	25.9	25.6	18.0	24.9
Separated/divorced	2.0	2.9	2.0	2.3
Widowed	0.3	0.3	0.0	0.1
Children (%)				
0	83.5	80.7	88.1	82.6
1	5.3	5.8	4.5	5.2
≥ 2	9.7	11.7	7.5	10.5

The authors assert that all procedures contributing to all work reported in this article complies with the ethical standards of the relevant national and institutional committees on human experimentation and with the Helsinki Declaration of 1975, as revised in 2008. All participants gave informed consent. The study was approved by the University of Manchester research ethics committee. All members of the Faculty of Medical and Human Sciences at the University of Manchester received an emailed invitation to participate including undergraduates, postgraduates, academics, clerical and general staff, allowing a reasonably diverse age range. The study was also advertised through an online social networking site.

#### TEAQ Item Generation

Questionnaire items were produced via discussion with professional psychologists and psychiatrists. The main types of interpersonal positive touch were identified as hugs, kisses, skin–skin, and hair–skin contact. The main circumstances in which interpersonal positive touch occurs were identified as greeting, consoling, intimacy, and childhood contact. Circumstances relating to non-interpersonal positive touch were identified as self-care, including personal grooming behaviours, touch with animals, and touching fabrics. Questionnaire items were systematically generated to cover the main types of touch and circumstances in which touch occurs. For each circumstance and touch type, 3 items were generated where possible: one to determine how often an individual experiences that touch, one to determine the attitude an individual has towards receiving that touch, and one to determine the attitude an individual has of giving that touch to someone else. Some general questions about attitude to and experience of touch were also included, such as: “I am put off by physical familiarity” and “My life lacks physical affection.” Items consisted of statements and a 5-point Likert scale of agreement, with “*Disagree strongly*” being the point furthest on the left and “*Agree strongly*” being the point furthest on the right. The following values were assigned to responses: 1 = “*Disagree strongly*”, 2 = “*Disagree a little*”, 3 = “*Neither agree nor disagree*”, 4 = “*Agree a little*”, 5 = “*Agree strongly*”, apart from negatively worded items which were reverse scored.

The terms ‘partner’, ‘boyfriend/girlfriend’, ‘husband/wife’ were avoided and instead any questions about intimate touch referred to ‘someone you are close to/fond of/know intimately’. Questions about childhood touch were limited to the amount of various forms of touch they recalled receiving and not their attitudes, as the latter was considered harder to recall and interpret. 117 items were generated. Item order was randomized using a random number generator. The item order remained the same for all participants.

#### Procedure

Participants completed the TEAQ online alongside some general questions to obtain demographic data. This investigation was carried out online, allowing anonymity, wider dissemination, and minimal influence of embarrassment, social conformity, and pressure to participate. The first sample was for item reduction and selection.

#### Principal Component Analysis (PCA) and Item Selection

PCA, rather than exploratory factor analysis was carried out on the initial dataset from the 117 item touch questionnaire, as we wanted to use a data reduction technique, to reduce the number of variables in the questionnaire while retaining as much information as possible, rather than simply identifying the latent constructs underlying the questionnaire variables (Fabrigar et al. 1999). PCA with direct oblimin rotation was carried out using SPSS Version 16 (SPSS Inc., Chicago, IL). Covariance rather than correlations analysis was used because covariance analysis is less influenced by variation in the distribution of scores between items on a 5-point Likert scale (Field 2005; Tinsley and Tinsley 1987). Missing values were excluded pairwise. Responses for negatively phrased items were reverse scored, so all item scores reflected greater touch experience or positive attitude. The number of components extracted was determined using Cattell’s scree test (Cattell 1966).

A correlation matrix was used to exclude redundant items correlating significantly with > 80% of other items, or correlating *r* > 0.8 with another item. Items with measures of sampling adequacy (MSA) < 0.6 were removed, as were items with rescaled communalities < 0.3, indicating these items explained only a small proportion of shared variance ( Field 2005). Stevens (1992) suggests component loadings > 0.4 should be considered of interest. Any items with component loadings < 0.4 for all components were removed, as were any items loading similarly on two components. Reliability analysis was carried out for each component, for which all items belonging to each component were given an equal weighting. Items which did not significantly increase Cronbach’s α were removed. For the remainder of this manuscript, subscale scores have been calculated by reverse scoring the items indicated with an **R** in Table 2, then calculating a mean score for each subscale.

**Table 2:** TEAQ component structure

	Component					
	FFT (29.2)	CIT (7.9)	ChT (6.3)	ASC (5.6)	AIT (4.1)	AUT (3.3)
I always greet my friends and family by giving them a hug (Q30)	0.75					
I often link arms with my friends and family as I walk along (Q57)	0.74					
I like to link arms with my friends and family as I walk along (Q13)	0.71					
I usually hug my family and friends when I am saying goodbye (Q14)	0.70					
I find it natural to greet my friends and family with a kiss on the cheek (Q4).	0.70					
I often make physical contact with my friends and family when I am with them (Q38)	0.69					
It’s nice when friends and family members greet me with a kiss (Q16)	0.64					
I am on huggable terms with quite a few people (Q48)	0.63					
I regularly hug people I am close to (Q21)	0.62					
I like it when my friends and family greet me by giving me a hug (Q56)	0.61					
I often put my arm around a close friend as we walk along together (Q51)	0.58					
Most days I get a hug or a kiss (Q36)		− 0.79				
I often share a romantic kiss (Q41)		− 0.79				
I often have sex (Q27)		− 0.75				
I don’t get many hugs these days (Q53 **R**)		− 0.75				
My life lacks physical affection (Q23 **R**)		− 0.75				
I often hold hands with someone I am fond of (Q46)		− 0.68				
I often fall asleep while holding someone I am close to (Q49)		− 0.67				
I often have my skin stroked (Q45)		− 0.62				
I often snuggle up on the sofa with someone (Q11)		− 0.60				
I can always find somebody to physically comfort me when I am upset (Q29)		− 0.58				
I often take a shower or bath with someone (Q25)		− 0.56				
I am often given a shoulder massage (Q54)		− 0.55				
When I am upset, there is usually someone who can comfort me (Q18)		− 0.51				
I often hold hands with someone I know intimately (Q17)		− 0.43				
As a child my parents would tuck me up in bed every night and give me a hug and a kiss goodnight (Q22)			− 0.80			
My parents were not very physically affectionate towards me during my childhood (Q9 **R**)			− 0.80			
There was a lot of physical affection during my childhood (Q5)			− 0.78			
As a child my parents always comforted me when I was upset (Q33)			− 0.73			
As a child I would often hug family members (Q6)			− 0.72			
As a child my parents would often hold my hand when I was walking along with them (Q35)			− 0.66			
As a child I found a hug from my parents when I was upset made me feel much happier (Q15)			− 0.65			
My mother regularly bathed me as a child (Q32)			− 0.57			
As a child my mother regularly brushed my hair (Q42)			− 0.53			
I like to use face masks on my skin (Q55)				− 0.77		
I like to use bath essence when having a bath (Q7)				− 0.76		
I like having a bath with lots of bubble bath (Q52)				− 0.70		
I like exfoliating my skin (Q43)				− 0.67		
I like using body lotions (Q2)				− 0.64		
I like to stroke the skin of someone I know intimately (Q47)					0.74	
I enjoy the feeling of my skin against someone else’s if I know them intimately (Q34)					0.69	
Its feels really good when someone I am fond of runs their fingers through my hair (Q20)					0.69	
I find stroking the hair of a person I am fond of very pleasurable (Q8)					0.67	
I enjoy having my skin stroked (Q24)					0.67	
Snuggling up on the sofa with someone is great (Q50)					0.62	
I enjoy being cuddled by someone I am fond of (Q31)					0.59	
I enjoy holding hands with someone I am fond of (Q40)					0.59	
I like to fall asleep in the arms of someone I am close to (Q10)					0.59	
Kissing is an enjoyable part of expressing romantic feeling (Q44)					0.56	
I enjoy the physical intimacy of sexual foreplay (Q12)					0.53	
Kissing is a great way of expressing physical attraction (Q19)					0.51	
I enjoy having sex (Q26)					0.47	
It makes me feel uncomfortable if someone I don’t know very well touches me in a friendly manner (Q39 **R**)						0.79
If someone I don’t know very well puts a friendly hand on my arm it makes me feel uncomfortable (Q37 **R**)						0.79
I have to know someone quite well to enjoy a hug from them (Q3 **R**)						0.71
I am put off by physical familiarity (Q28 **R**)						0.45
I dislike people being very physically affectionate towards me (Q1 **R**)						0.44

Component structure of the 57-item Touch Experiences and Attitudes Questionnaire (TEAQ). The final component structure and the component loading of each item are shown. Item numbers are shown in parentheses after each item, with an **R** where appropriate to denote reverse scored items. Component 1: friends and family touch (FFT), Component 2: current intimate touch (CIT), Component 3: childhood touch (ChT), Component 4: attitude to self-care (ASC), Component 5: attitude to intimate touch (AIT), Component 6: attitude to unfamiliar touch (AUT). Numbers in parentheses after the component names represent the percentage variance explained by each component

### Results

#### Principal Component Analysis (PCA)

Cattell’s scree test identified 6 components. A total of 60 items failed inclusion criteria: 40 had low communality scores, 7 loaded < 0.4 on all components, 6 had low MSA scores, 3 did not increase Cronbach’s α, 1 loaded similarly on two components, 1 significantly correlated with over 80% of the other questionnaire items and 2 pairs of items correlated with each other > 0.8, so one item from each pair was deleted. This left a total of 57 items explaining 56% of the variance. The component structure identified is presented in Table 2. A copy of the TEAQ with scoring instructions can be found in “Appendix 1”. Copyright of the TEAQ remains with the authors. The range of Cronbach’s α for the components was 0.78–0.92, suggesting one dimension per component and internal consistency.

Of the components identified, touch with others concerned 5 components and one component (component 4) concerned attitude to self-care (5 items). The largest component in terms of variance explained, contained 11 items and was termed Friends and Family Touch (FFT) because it loaded on items about amount and liking of giving and receiving affectionate touch with friends and family. This appears to be a general component since it correlated with other component scores *r* = 0.50–0.52, except attitude to self-care where *r *= 0.36. The FFT component contains both attitudinal and amount measures, but as these all loaded on the same component, it appears these measures cannot be separated for this component. Two components (2 and 3) concerned amount of touch, respectively in intimate relationships (14 items) and in childhood (9 items), and two (5 and 6) concerned affective attitude to touch with others, respectively in intimate relationships (13 items) and unfamiliar touch (5 items). Component names are shown in Table 2.

#### Component Correlations

As shown in Table 3, component scores correlated significantly with each other (*p *< 0.001). For this reason, the solution obtained from direct oblimin, oblique rotation was accepted, rather than an orthogonally rotated solution (Tabachnick and Fidell 2013). Attitude to self-care correlated least with other components and friends and family touch correlated most. The strongest correlation was between current intimate touch and attitude to intimate touch (*r* = 0.58). The weakest correlation was between attitude to self-care and attitude to unfamiliar touch (*r* = 0.12).

**Table 3:** TEAQ component correlations with each other

	FFT	CIT	ChT	ASC	AIT	AUT
Friends and family touch (FFT)	1.00	.50	.52	.36	.51	.51
Current intimate touch (CIT)	.50	1.00	.41	.30	.58	.30
Childhood touch (ChT)	.52	.41	1.00	.25	.35	.28
Attitude to self-care (ASC)	.36	.30	.25	1.00	.28	.12
Attitude to intimate touch (AIT)	.51	.58	.35	.28	1.00	.41
Attitude to unfamiliar touch (AUT)	.51	.30	.28	.12	.41	1.00

All correlations are significant at *p* < 0.001

### Discussion

This study identified a 6-component structure: one component related to interpersonal physical touch experiences and attitudes with friends and family, named friends and family touch (FFT). The second component related to current levels of intimate touch experienced (Current Intimate Touch, CIT), relating to touch usually experienced between people who are emotionally close or in a romantic relationship. The third component related to positive childhood touch experiences (Childhood Touch, ChT). The fourth component related attitude to self-care (ASC), relating to how much individuals liked various skin care and grooming behaviours relating to positive self-care. The fourth component relates to attitudes to intimate touch (AIT), relating to how much individuals enjoy touch experiences which usually occur between individuals who are emotionally close or in a romantic relationship. These experiences are comparable to those referred to in the current intimate touch component. Finally, the last component, attitude to unfamiliar touch (AUT), relates to how comfortable people are with physical touch received from people the individual is less close to, including interpersonal touch from people who are not family, friends or those emotionally close to the individual.

It is interesting to note that the only non-interpersonal touch items that were not eliminated from the questionnaire were those relating to self-care, with items relating to stroking animals and touching fabrics not meeting criteria for questionnaire inclusion. Self-care is clearly of importance and relevance. It is interesting that those with severe mental health difficulties may neglect their physical appearance and personal hygiene, reflecting a neglect in self-care (Corrigan 2000; First et al. 2002; Häfner et al. 2003). Further investigation of the relationship between this self-care measure and mental health would be of interest.

The components identified provide a distinction between touch experienced between friends and family, those known more intimately and those not well known, highlighting the context in which physical touch is experienced is of importance, a result supported by fMRI studies such as those of McCabe et al. (2008) and Gazzola et al. (2012) where manipulating the context in which touch was experienced significantly altered the central responses to touch induced. Although the component structure of the TEAQ appeared good in terms of internal consistency and face validity, further validation of the component structure was required, as detailed in Study 2.

## Study 2

### Introduction

Although the face validity of the components identified in study 1 appeared to be good and the structure identified can be supported by prior literature into positive touch experiences, it was necessary to determine whether this factor structure was valid using confirmatory factor analysis and data collected from a second sample of participants.

### Method

#### Participants

The sample for this study was recruited in the same way as that described for Study 1. The questionnaire battery, including the 57-item TEAQ was completed by 817 participants. Of these participants, 113 were excluded due to > 5% missing data, leaving 704 participants, 73.7% of which were female. Mean ± standard deviation age was 27.4 ± 9.6 years. As shown in Table 1, socio-economic demographics were similar to the study 1 sample.

#### Confirmatory Factor Analysis (CFA)

The replication sample completed the reduced TEAQ online and CFA was carried out using AMOS statistical software (Amos™ 7; SPSS Inc.). The criteria used to determine goodness of model fit were a Root Mean Square Error of Approximation (RMSEA) < 0.06 with a narrow confidence interval, an RMSEA probability value > 0.5, a Comparative Fit Index (CFI) > 0.95, a low Akaike’s Information Criterion (AIC), a standardised Root Mean Square Residual (RMR) < 0.05 and a Tucker-Lewis Index (TLI) > 0.95 (Byrne 2001).

### Results

The 6-component structure previously identified by PCA provided a reasonable fit of the CFA sample data (Table 4). The reliability of structural equation modeling is reduced by an excessively large number of variables. It was therefore determined whether parcellation of items to produce three measures for each component affected the outcome, as advocated by Yang et al. (2010), using the procedure of Nasser and Wisenbaker (2003). For each parcel, a mean score for the items belonging to that parcel was calculated. The number of items per parcel was determined by dividing the number of items contained in each component by 3, allowing parcels per component to contain as close to an equal number of items as possible. The items contained in each parcel were chosen so that if the pattern matrix identified from PCA was valid in the CFA sample, each parcel would load onto its latent variable equally, as lowest and highest loading items were parcelled together. Due to the unequal number of items per component, some parcels contained 5 items, whereas other items were not parcelled and treated as individual variables. While this does cause some unequal loading of items when the overall structure of the questionnaire is considered, parcellation per component was designed so that all items loaded similarly on their latent variable. The aim of CFA in this investigation was to determine whether or not the components previously identified were valid. It can be seen in Table 4 that parcellation improved model fit, with all criteria for a good model fit being met. The models used for CFA, including the items belonging to each parcel, have been provided in Online Supplementary Materials (Figures S1 and S2).

**Table 4:** Model fit indices for the TEAQ models tested using confirmatory factor analysis

	Goodness of fit tests (criterion value)					
	RMSEA (< .06), 90% CI	RMSEA *p* (> 0.5)	CFI (> .95)	AIC	Std RMR (< .05)	TLI (> .95)
Original model	0.069, 0.067–0.070	< 0.001	0.805	6811	0.071	0.796
Parcelled model	0.055, 0.049–0.062	0.081	0.974	480	0.034	0.967

*RMSEA* Root mean square error of approximation, *CI* confidence interval, *RMSEA p* probability statistic associated with the RMSEA, *CFI* comparative fit index, *AIC* Akaike’s information criterion, *Std RMR* standardised root mean square residual, *TLI* Tucker-Lewis Index

### Discussion

Confirmatory factor analysis confirmed a 6-factor structure of the TEAQ in a second sample of participants. An appropriate use of the TEAQ is therefore to calculate scores for each subscale to investigate participants’ positive touch experiences and attitudes.

## Study 3

### Introduction

The third study was conducted to examine the criterion-related validity of the TEAQ in terms of concurrent and predictive validity. To examine predictive validity, the TEAQ was completed alongside the short form of the Childhood Trauma Questionnaire (CTQ-SF) (Bernstein et al. 2003), as the TEAQ childhood touch (ChT) subscale was expected to be negatively predictive of childhood trauma. The Social Support Questionnaire (SSQ6) (Sarason et al. 1987) was also included as the TEAQ factors relating to current interpersonal touch experience subscales in particular, friends and family touch (FFT) and current intimate touch (CIT) were expected to predict current levels of perceived social support.

To determine concurrent and discriminant validity of the TEAQ, the 57-item TEAQ was completed alongside other physical touch questionnaires which are currently available. Examination of the literature identified seven physical touch questionnaires to potentially include in this study. These questionnaires were the Touch Avoidance Measure (TAM) (Andersen and Leibowitz 1978), the Familial Touch Orientation (FTO) scale (Gladney and Barker 1979), the TACTYPE questionnaire (Deethardt and Hines 1983), the Touch Test (Fromme et al. 1989), the Questionnaire on Physical Contact Experience (QPCE) (Cochrane 1990), the Physical Contact Assessment Questionnaire (Weiss et al. 2000), and the Social Touch Questionnaire (STQ) (Wilhelm et al. 2001).

Three of these questionnaires the TAM, TACTYPE questionnaire, and the Touch Test were deemed rather dated and unsuitable for modern use, with items heavily focused on attitudes to same versus opposite sex touch and some items being very specific in nature. Example questions include, “When I see two people of the same sex hugging, it revolts me,” (item 4, TAM), “When I tell a same-sex intimate friend I have just gotten a divorce, I want that person to touch me,” (item 2, TACTYPE questionnaire) and “How comfortable would you feel hugging a same-sex person who was homely?” (item 1, the Touch Test). The authors attempted to include the Physical Contact Assessment Questionnaire (Weiss et al. 2000), but were unable to obtain a copy from the authors.

### Method

#### Participants

This study was approved by Liverpool John Moores Research Ethics Committee. Participants were recruited from Liverpool John Moores University. Both students and staff were invited to take part. In total, 210 participants took part in this study. Any participants with any missing responses for any questionnaire were excluded to minimize inaccuracies caused by missing values (Tabachnick and Fidell 2007), resulting in the exclusion of 9 participants. The final study sample consisted of 201 participants of which 79.6% were female. Participants were aged 18–81 years old, mean ± standard deviation age was 25.5 ± 10.1 years. As shown in Table 1, socio-economic demographics were similar to Studies 1 and 2, although the sample consisted of more students and fewer participants who were working full-time.

#### Procedure

Participants completed the TEAQ as part of an online questionnaire battery. The questionnaire battery additionally included some general questions to obtain demographic data. To investigate the concurrent and discriminant validity of the TEAQ compared to previously published physical touch questionnaires, three additional physical touch questionnaires were included in the questionnaire battery. The 16-item Familial Touch Orientation (FTO) Scale (Gladney and Barker 1979) is a measure of positive physical touch experiences as a child. A low score on this scale represents a high frequency of positive touch experiences during childhood. The Social Touch Questionnaire (Wilhelm et al. 2001) is a 20-item questionnaire focusing on attitudes to physical touch. A low score on this questionnaire represents a more positive attitude towards social touch.

The final physical touch questionnaire included, the Questionnaire on Physical Contact Experience (QPCE) (Cochrane 1990) is an 8-item questionnaire which asks participants to rate on a four-point scale from “*None*” to “*A lot*”, how much good, bad and ‘other’ physical contact they experienced during childhood and at present. Two further items ask whether participants believe they were loved as a child and at present. The QPCE produces two dichotomous variables, one for childhood physical contact experience and another for present physical contact experience. Satisfactory physical contact experience, represented by a score of 1, is defined as the experience of substantial good physical contact with no substantial bad physical contact. If these conditions are not met, a score of 0, representing unsatisfactory physical contact experience is given.

To investigate the predictive validity of the TEAQ, the 28-item short form of the Childhood Trauma Questionnaire (CTQ-SF) (Bernstein et al. 2003) was included, as the TEAQ ChT subscale was expected to be negatively predictive of childhood trauma. The CTQ contains five items per subscale, with three additional minimisation questions. The five subscales of this questionnaire relate to childhood sexual, physical and emotional abuse and physical and emotional neglect. Analysis of the factor structure of the childhood trauma questionnaire has identified that using the total score, excluding the 3 minimisation items, is a good fit of the data and a valid use of the questionnaire (Spinhoven et al. 2014), therefore a total CTQ score was calculated and included the analysis.

The 6-item Social Support Questionnaire (SSQ6) (Sarason et al. 1987) was additionally included, as TEAQ subscales relating to current interpersonal touch experience in particular were expected to predict current social support. The SSQ6 was altered slightly. The first question: ‘‘Whom can you really count on to distract you from your worries when you feel under stress?” was replaced with a similar item from the original Social Support Questionnaire (SSQ) (Sarason et al. 1983): “Whom can you really count on to be dependable when you need help?”. It was felt that this item was easier to understand, more general, and more relevant, allowing a wider range of situations to be covered. The SSQ6 was also simplified by asking participants to rate the number of people they could depend on for each circumstance as “*None*” “*A Few*” or “*Lots*” rather than asking participants to name the people they could depend on in each circumstance. As with the original SSQ6, participants rated both the number of people they could depend on and their satisfaction with the support they currently experienced for each item, allowing two scores to be produced; number of social supports (SSQN) and satisfaction with social support (SSQS).

This investigation was carried out online using Qualtrics^®^ software (Qualtics, Provo, UT), allowing anonymity, wider dissemination and minimal influence of embarrassment, social conformity and pressure to participate. Demographic questions were always completed first, but the remaining questionnaires were completed in a randomised order, except for the FTO, which was always completed last. This is because the FTO defines childhood as birth to 10 years of age, but a cut-off age for childhood for the other two childhood touch measures, the QPCE and the TEAQ ChT scales was deliberately left unspecified (Cochrane 1990). Therefore, to ensure consistency between participants, the FTO was always completed last. After completing these questionnaires, participants were thanked for their participation and provided with a debriefing sheet.

#### Statistical Analysis

All questionnaires were scored according to authors’ instructions and all data analyses were carried out using IBM^®^ SPSS^®^ version 23. Recruitment was not targeted towards participants who had experienced childhood trauma. As such, the majority of participants had very low scores on the CTQ subscales, so this data was not normally distributed. For this reason, bootstrapping was used for multiple regression analysis and Spearman’s rho correlation coefficients are reported.

### Results

As seen in Table 5, convergent validity of the TEAQ factors is good, with factor scores correlating as expected with the other physical touch measures. The TEAQ childhood touch measure correlated strongly with the familial touch orientation (FTO) scale (*r*_*S*_ = − .76, *p* < .001) and the QPCE childhood measure (*r*_*S*_ = .51, *p* < .001). As expected, the QPCE present measure correlated most strongly with the current intimate touch (CIT) factor of the TEAQ (*r*_*S*_ = .56, *p* < .001). The social touch questionnaire correlated moderately to strongly with all TEAQ factors, apart from the attitude to self-care (ASC) factor score. Interestingly, the STQ correlated most strongly with the attitude to unfamiliar touch factor of the TEAQ (*r*_*S*_ = − .74, *p* < .001), although as the STQ was designed for a study investigating anxiety towards social touch situations, including situations involving touch with strangers, this is not particularly surprising.

**Table 5:** Spearman’s correlations of all social touch measures included in the study

Spearman’s rho	FTO	QPCE.C	QPCE.P	STQ
TEAQ.ChT	− .76***	.51***	.24***	− .42***
TEAQ.FFT	− .50***	.35***	.26***	− .67***
TEAQ.CIT	− .33***	.17*	.56***	− .43***
TEAQ.ASC	− .26***	.30***	0.12	− .16*
TEAQ.AIT	− .25***	0.12	.38***	− .51***
TEAQ.AUT	− .28***	0.11	.23***	− .74***

*TEAQ* Touch Experiences and Attitudes Questionnaire, *ChT* childhood touch, *FFT* friends and family touch, *CIT* current intimate touch, *ASC* attitude to self-care, *AIT* attitude to intimate touch, *AUT* attitude to unfamiliar touch, *FTO* familial touch orientation scale, *QPCE.C* questionnaire on physical contact experience childhood score, *QPCE.P* questionnaire on physical contact experience present score, *STQ* social touch questionnaire

***p ≤ 0.001; **p ≤ 0.01; *p ≤ 0.05 (2-tailed). Correlation coefficients > .3 are indicated in bold

Additionally, in Table 6, it can be seen that, out of all physical touch measures used in this study, total childhood trauma correlates most strongly with the TEAQ Childhood Touch (ChT) measure (*r*_*S*_ = − .65, *p* < .001). Out of the childhood trauma and social support measures used in this study, TEAQ ChT and FTO scores correlated most strongly with childhood emotional neglect (*r*_*S*_ = − .72 and .65 respectively, *p* < .001) and QPCE childhood correlated most strongly with total childhood trauma score (*r*_*S*_ = − .51, *p* < .001) and correlated equally strongly with childhood emotional neglect and physical abuse (*r*_*S*_ = .49, *p* < .001). Out of the social touch measures used, number of social contacts (SSQN) correlated most strongly with FTO score (*r*_*S*_ = − .41, *p* < .001) and satisfaction with social support correlated most strongly with TEAQ current intimate touch (CIT) score (*r*_*S*_ = .46, *p* < .001).

**Table 6:** Spearman’s correlations of all social touch measures with Childhood Trauma and social support measures

Spearman’s rho	CTQ emotional neglect	CTQ emotional abuse	CTQ physical neglect	CTQ physical abuse	CTQ sexual abuse	Total CTQ	SSQN	SSQS
TEAQ.ChT	− .72***	− .49***	− .43***	− .33***	− .21**	− .65***	.38***	.35***
FTO	.65***	.52***	.38***	.27***	.12	.60***	− .41***	− .32***
QPCE.C	− .49***	− .35***	− .43***	− .49***	− .29***	− .51***	.26***	.23***
TEAQ.FFT	− .44***	− .29***	− .30***	− .16*	− .01	− .38***	.35***	.26***
TEAQ.CIT	− .32***	− .26***	− .21**	− .17*	− .05	− .31***	.36***	.46***
TEAQ.ASC	− .21**	− .10	− .19**	− .08	− .08	− .17*	.19**	.15*
TEAQ.AIT	− .16*	− .09	− .18**	− .03	− .01	− .16*	.30***	.24***
TEAQ.AUT	− .22**	− .24***	− .18**	− .03	− .16*	− .22***	.24***	.14*
STQ	.35***	.29***	.28***	.11	.05	.34***	− .35***	− .27***
QPCE.P	− .25***	− .26***	− .15*	− .04	.004	− .24***	.36***	.42***

*TEAQ* Touch Experiences and Attitudes Questionnaire, *ChT* childhood touch, *FFT* friends and family touch, *CIT* current intimate touch, *ASC* attitude to self-care, *AIT* attitude to intimate touch, *AUT* attitude to unfamiliar touch, *FTO* familial touch orientation scale, *QPCE.C* questionnaire on physical contact experience childhood score, *QPCE.P* questionnaire on physical contact experience present score, *STQ* social touch questionnaire, *CTQ* childhood trauma questionnaire, *SSQN* number of social contacts, *SSQS* satisfaction with social support

***p  ≤  0.001; **p  ≤  0.01; *p  ≤  0.05 (2-tailed). Correlation coefficients > .3 are indicated in bold

#### Reliability Analysis

For all measures used in this study, Cronbach’s alpha was good. The scale with the lowest Cronbach’s alpha was the CTQ physical neglect scale (α = .77) and the highest being for the CTQ sexual abuse scale (α = .96). For the TEAQ subscales, the current intimate touch subscale had the highest Cronbach’s alpha (α = .93) and the attitude to self-care subscale had the lowest Cronbach’s alpha (α = .81).

Cronbach’s alpha for the STQ was .88. interestingly, item number 20 “I like petting animals” had an extremely low correlation with the total STQ score (*r* = .06). This is the only item relating to touch with animals. STQ item number 12, “As a child, I was often cuddled by family members (e.g., parents, siblings)”, the only item relating to physical touch during childhood and item number 18, “If I had the means, I would get weekly professional massages” also correlated < .3 with total STQ score (*r* = .25 and .26 respectively), suggesting these items are not strongly related to the underlying construct the STQ is measuring. As the factor structure of the STQ is unknown, this is not necessarily surprising.

For all other measures used in this study, all items correlated with total measure scores > .3, except for the FTO scale item number 2 “Wrestled with brothers/sisters or parent(s)”, where *r* = .23.

### Multiple Regression Analyses

#### Total Childhood Trauma

A robust hierarchical regression analysis based on 1000 bootstrap samples was carried out using the total CTQ score. All assumptions for this analysis, as described by Field (2013) were met. Although there were no issues with multicollinearity, the familial touch score correlated highly with the TEAQ childhood touch score (*r* = − .77). To understand the predictive validity of these two measures, these measures were entered into the regression model separately, to understand whether one measure explained significantly more of the variance in total CTQ score than the other. TEAQ childhood touch was entered into the model first and significantly predicted CTQ total score (*F*_1,199_ = 128.30, *p* < .001), explaining 39.2% of the variance. Addition of FTO total score did not explain significantly more of the variance in CTQ total score (*ΔR*^2^ = .01, *ΔF*_1,198_ = 2.28, *p* = .133).

When FTO was entered into the model first, FTO significantly predicted total CTQ score, explaining 28.5% of the variance (*F*_1,199_ = 79.21, *p* < .001), but addition of TEAQ ChT score explained significantly more of the variance than FTO score alone (*ΔR*^2^= .11, *ΔF*_1,198_ = 37.62, *p* < .001) and when TEAQ ChT score was added, FTO score was no longer significantly predictive of CTQ total score (*β* = 0.13, *p* = .141). The predictive validity of TEAQ ChT for CTQ total score appears to be greater than FTO score. Addition of QPCE childhood score to this model explained significantly more of the variance (*ΔR*^2^ = .08, *ΔF*_1,197_ = 31.03, *p* < .001), this is likely due to QPCE childhood score taking into account negative as well as positive touch experienced during childhood, so explains some of the variance not explained by either FTO score or TEAQ ChT score. However, when QPCE childhood was entered into the model first, it was significantly predictive of total CTQ score, explaining 35.3% of the variance (*F*_1,199_ = 108.627, *p* < .001), but addition of TEAQ ChT explained significantly more of the variance (*ΔR*^2^= 0.120, *ΔF*_1,198_ = 44.991, *p* < .001). This highlights the discriminant validity of the TEAQ ChT scale in that the variance explained by all three childhood touch measures is not entirely conflated. Addition of all adult touch measures did not explain significantly more of the variance than the childhood touch measures alone (*ΔR*^2^ = .03, *ΔF*_7,190_ = 1.59, *p* = .142).

Overall, the model with the 3 childhood touch predictors explained 48.1% (*R*^2^ = .48) of the variance in total CTQ score with adjusted *R*^2^ being very similar (adjusted *R*^2^ = .47), suggesting the model to be generalizable. Results are presented in Table 7.

**Table 7:** Linear model of predictors of total CTQ score, with 95% bias corrected and accelerated confidence intervals reported in parentheses. Confidence intervals and standard errors based on 1000 bootstrap samples

	*B*	*SE* *B*	*β*	*p*
Model 1				
Constant	6.45 (− 0.27, 12.18)	3.34		.060
FTO	1.51 (1.17, 1.90)	0.19	.53	.001
Model 2				
Constant	57.08 (36.81, 78.57)	10.75		.001
FTO	0.37 (− 0.14, 0.87)	0.25	.13	.141
TEAQ ChT	− 7.13 (− 10.85, − 3.98)	1.61	− .53	.001
Model 3				
Constant	53.75 (35.91, 75.13)	9.90		.001
FTO	0.39 (− 0.05, 0.78)	0.22	.14	.069
TEAQ ChT	− 4.30 (− 7.69, − 1.53)	1.55	− .32	.008
QPCE childhood	− 11.07 (− 16.31, − 5.83)	2.38	− .35	.001

Model 1, *R*^2^ = .29, *p *= < .001; Model 2; *ΔR*^2^ = .11, *p* = < .001, Model 3, *ΔR*^2^ = .08, *p* < .001

*TEAQ* Touch Experiences and Attitudes Questionnaire, *ChT* childhood touch, *FTO* familial touch orientation scale, *QPCE* questionnaire on physical contact experience

#### Multiple Regression Analysis to Determine Which CTQ Factors are Most Predictive of TEAQ ChT Score

To understand the relationship between TEAQ ChT score and childhood trauma, a robust hierarchical regression analysis based on 1000 bootstrap samples was carried out to determine which childhood trauma factor was most predictive of TEAQ ChT score. We had no a priori hypotheses about which childhood trauma factors we expected to be most predictive of TEAQ ChT score, so all predictors were entered into the model simultaneously. All assumptions for this analysis, as described by Field (2013) were met.

Overall, the model significantly predicted TEAQ ChT score (*F*_5,195_ = 60.78, *p* < .001), explaining 60.9% (*R*^2^ = .61) of the variance, with adjusted *R*^2^ being similar (adjusted *R*^2^ = .60), suggesting the model to be generalizable. Childhood emotional neglect was significantly negatively predictive of TEAQ ChT (*β* = − .85, *p* = .001). The other childhood trauma factors were not significantly predictive of TEAQ ChT score. Results are presented in Table 8.

**Table 8:** Linear model of childhood trauma questionnaire (CTQ) predictors of TEAQ childhood touch (ChT) score, with 95% bias corrected and accelerated confidence intervals reported in parentheses. Confidence intervals and standard errors based on 1000 bootstrap samples

	*B*	*SE B*	*β*	*p*
Constant	5.48 (5.23, 5.68)	0.13		.001
Emotional neglect	− 0.19 (− 0.23, − 0.16)	0.02	− .85	.001
Emotional abuse	0.01 (− 0.03, 0.05)	0.02	.04	.638
Physical neglect	0.01 (− 0.04, 0.06)	0.03	.02	.742
Physical abuse	0.03 (− 0.02, 0.07)	0.02	.07	.282
Sexual abuse	− 0.01 (− 0.05, 0.06)	0.03	− .03	.640

*R*^2^ = .61, *p *= < .001

#### Satisfaction with Social Support (SSQS)

A hierarchical robust regression analysis based on 1000 bootstrap samples was carried out using satisfaction with social support as the outcome variable. All assumptions for this analysis, as described by Field (2013) were met. To investigate the predictive validity of the TEAQ, all TEAQ factor scores were entered into the model, then it was determined whether the touch scores from the other questionnaire measures explained significantly more of the remaining variance. If not, then the other questionnaire measures would not be more predictive of satisfaction with social support than the TEAQ. It was expected that out of the TEAQ measures, the factors relating to current touch experience would be more predictive of satisfaction with social support than the factors relating to attitude to physical touch and that all factors relating to current attitudes and experiences of physical touch would be more predictive of satisfaction with social support than the childhood touch measure. For these reasons, the two factors relating to current touch experience, FFT and CIT were added to the model in the first block, followed by the TEAQ attitude factors, ASC, AIT and AUT in the second block. The third block contained the TEAQ ChT factor. The remaining touch measures were then added. Out of these measures, it was again predicted those relating to current touch experience would be more predictive of satisfaction with social support than those relating to childhood touch experiences, so the two factors relating to current touch experience, QPCE present an STQ were entered in the fourth block followed by QPCE childhood and FTO total in the fifth block. All assumptions for this analysis, as described by A. Field (2013) were met.

The two TEAQ factors relating to current touch, FFT and CIT were entered into the model first and explained a significant amount of the variance in satisfaction with social support (*F*_2,198_ = 25.99, *p* < .001), explaining 20.8% of the variance. Addition of the TEAQ measures relating to attitudes to physical touch, ASC, AIT and AUT, explained significantly more of the variance in satisfaction with social support (*ΔR*^2^ = .06, *ΔF*_3,195_ = 5.17, *p* = .002). Addition of the TEAQ ChT measure explained significantly more of the variance (*ΔR*^2^ = .02, *ΔF*_1,194_ = 4.19, *p* = .042). Addition of the two remaining current touch measures QPCE present and STQ, explained significantly more of the variance (*ΔR*^2^ = .03, *ΔF*_2,192_ = 4.54, *p* = .012). Addition of the remaining childhood touch measures, QPCE childhood and FTO, did not explain significantly more of the variance (*ΔR*^2^ = .01, *ΔF*_2,190_ = 1.22, *p* = .298).

As the addition of the remaining childhood touch measures, QPCE childhood and FTO, did not explain significantly more of the variance, these measures were removed from the model. The final results for this analysis are presented in Table 9.

**Table 9:** Linear model of predictors of satisfaction with social support, with 95% bias corrected and accelerated confidence intervals reported in parentheses. Confidence intervals and standard errors based on 1000 bootstrap samples

	*B*	*SE B*	*β*	*p*
Model 1				
Constant	2.94 (2.27, 3.54)	0.32		.001
TEAQ FFT	0.09 (− 0.10, 0.28)	0.10	.07	.358
TEAQ CIT	0.50 (0.33, 0.68)	0.09	.42	.001
Model 2				
Constant	3.70 (2.82, 4.51)	0.41		.001
TEAQ FFT	0.10 (− 0.10, 0.31)	0.10	.08	.338
TEAQ CIT	0.73 (0.48, 1.00)	0.14	.61	.001
TEAQ ASC	0.13 (− 0.03, 0.29)	0.08	.12	.103
TEAQ AIT	− 0.52 (− 0.86, − 0.21)	0.16	− .34	.004
TEAQ AUT	0.04 (− 0.15, 0.20)	0.09	.03	.685
Model 3				
Constant	3.39 (2.44, 4.28)	0.45		.001
TEAQ FFT	− 0.002 (− 0.22, 0.22)	0.11	− .001	.988
TEAQ CIT	0.70 (0.45, 0.99)	0.14	.58	.001
TEAQ ASC	0.08 (− 0.09, 0.27)	0.09	.07	.379
TEAQ AIT	− 0.49 (− 0.81, − 0.17)	0.16	− .31	.005
TEAQ AUT	0.05 (− 0.13, 0.23)	0.09	.04	.564
TEAQ ChT	0.19 (− 0.01, 0.39)	0.10	.16	.076
Model 4				
Constant	5.37 (3.25, 7.43)	1.07		.001
TEAQ FFT	− 0.08 (− 0.34, 0.17)	0.13	− .06	.535
TEAQ CIT	0.61 (0.37, 0.86)	0.14	.51	.001
TEAQ ASC	0.10 (− 0.08, 0.29)	0.09	.09	.240
TEAQ AIT	− 0.57 (− 0.90, − 0.25)	0.17	− .37	.002
TEAQ AUT	− 0.13 (− 0.35, 0.10)	0.12	− .10	.274
TEAQ ChT	0.16 (− 0.03, 0.37)	0.11	.14	.125
QPCE present	0.37 (− 0.15, 0.90)	0.25	.13	.143
STQ	− 0.02 (− 0.04, < 0.001)	0.01	− .25	.040

Model 1; *R*^2^ = .21, *p *= < .001; Model 2; *ΔR*^2^ = .06, *p* = .002, Model 3, *ΔR*^2^ = .02, *p *= .042, Model 4, *ΔR*^2^ = .03, *p *= .012

*TEAQ* Touch Experiences and Attitudes Questionnaire, *ChT* childhood touch, *FFT* friends and family touch, *CIT* current intimate touch, *ASC* attitude to self-care, *AIT* attitude to intimate touch, *AUT* attitude to unfamiliar touch, *QPCE* questionnaire on physical contact experience, *STQ* social touch questionnaire

Overall, the final model which included all 6 TEAQ factors and the QPCE present and STQ measures explained 31.4% (*R*^2^ = .31) of the variance in satisfaction with social support with adjusted *R*^2^ being similar (adjusted *R*^2^ = .29), suggesting the model to be generalizable. The final model identified the TEAQ CIT subscale as most predictive of satisfaction with social support (*β* = .51, *p* = .001), followed by the TEAQ AIT subscale (*β* = − .37, *p* = .002), followed by the STQ (*β* = − .25, *p* = .040). As expected, low levels of current intimate touch predicted lower satisfaction with social support. Additionally, those rating intimate touch more positively, in terms of TEAQ AIT score and those with a more negative attitude to social touch in general in terms of STQ score, with more anxiety and avoidance of touch situations, had lower satisfaction with social support.

### Discussion

This study has identified the convergent and predictive validity of the TEAQ to be good. TEAQ factor scores correlated as expected with the other physical touch measures, highlighting good convergent validity. Additionally, good predictive validity was identified, with the TEAQ ChT measure identified as having greater predictive validity than the FTO score for total childhood trauma. Discriminant validity of the TEAQ ChT factor was also identified, as the addition of the TEAQ ChT score after FTO score explained significantly more of the variance in total CTQ score.

The CTQ childhood emotional neglect score was significantly predictive of TEAQ ChT, highlighting the importance of the emotional component of positive physical touch, which CTs have been hypothesised to have a key role in encoding (Morrison et al. 2010). In terms of satisfaction with social support, the predictive validity of the TEAQ was demonstrated, explaining a significant amount of the variance in satisfaction with social support. Addition of the other current physical touch measures, the STQ and QPCE present scores explained a small, but significant amount of the remaining variance, highlighting the discriminant and differential predictive validity of the TEAQ, in that although the TEAQ, STQ, and QPCE present scores all relate to physical touch, the variance explained by these measures is not entirely conflated.

After identifying good convergent, predictive and discriminant validity of the TEAQ, the final study investigated known-group validity by investigating demographic differences in TEAQ responses.

## Study 4

### Introduction

Studies 1, 2, and 3 have identified the TEAQ to have good predictive validity and high internal consistency, as well as good construct validity in terms of convergent and discriminant validity. Our final study focused on identifying demographic differences in TEAQ responses to investigate construct validity in terms of known-group validity. We predicted there would be gender differences in TEAQ responses, with previous literature identifying females generally being more comfortable with interpersonal touch than males, particularly in terms of initiating touch (Webb and Peck 2015), so more positive attitudes to touch were predicted for females. Self-reported interpersonal touching behaviors in general have been identified to be greater in females than males (Jones 1986), suggesting self-reported interpersonal touching behaviours, as measured by the TEAQ FFT and CIT scales may be higher in females than males.

It has been previously identified that mothers engage in more positive touch with their daughters than sons (Goldberg and Lewis 1969; Lindahl and Heimann 1997; Robin 1982). Additionally, Takeuchi et al. (2010) identified a significant correlation between self-reported levels of positive parental touch experienced in early childhood and gender, suggesting greater levels of parental touch to be received by females than males. It was therefore predicted that females would report more positive parental touch in childhood than males, as measured by the TEAQ ChT subscale.

Self-reported attitudes to body care have been reported to be significantly higher in female adolescents than males (Brausch and Muehlenkamp 2007) and in adults use of personal care products and investment in appearance has been identified as greater in females than males (Biesterbos et al. 2013; Muth and Cash 1997). It was therefore predicted that scores on the TEAQ ASC subscale would be significantly greater for females than males.

Additionally, it was predicted that individuals in a romantic relationship or married would report higher levels of current intimate touch, as measured by the TEAQ CIT scale than those who were single. Additionally, it has been previously identified that touch is greatest in the intermediate stage of a relationship, compared to the beginning or stable stage (Guerrero and Andersen 1991), so it was predicted that TEAQ CIT scores would be greater for those in a relationship compared to those who were married or cohabiting.

The influence of age on TEAQ responses was also investigated, as previous studies have identified older individuals to be more comfortable with interpersonal touch (Webb and Peck 2015), so an increase in attitude to interpersonal touch with increasing age was predicted. In terms of touch initiation, Hall and Veccia (1990) identified no overall influence of age on touching behavior, however an interaction of age and gender was identified, with percentage of touch initiation in mixed sex dyads significantly decreasing with age for males and increasing for females, with no effect of age for same sex dyads. The influence of gender on the relationship between age and touch experiences and attitudes was therefore investigated.

### Method

The data from Studies 1, 2, and 3 were combined, then analyzed to investigate how gender, marital status, and age influence TEAQ subscale scores.

#### Participants

Before excluding participants due to missing data, the sample consisted of 1509 participants, of which 73.2% were female. Mean ± standard deviation age was 27.0 ± 9.6 years. Further demographic information for the sample is presented in Table 1.

#### Data Analysis

For all analyses, IBM^®^ SPSS^®^ version 23 was used. For the analysis of the effect of gender on TEAQ responses, independent samples *t* tests were used. Due to the very large sample size, Cohen’s *d* effect sizes were also calculated and effect sizes greater than .20 taken to be effects of interest (Cohen’s *d*: 0.2—small effect size; 0.5—medium effect size; 0.8—large effect size).

For the analysis of marital status, most participants could be categorized as single, in a relationship or married/cohabiting (Table 1), with similar numbers of participants per group. One-way ANOVAs were used to compare these three groups for the 6 TEAQ subscales. When Levene’s test was significant, meaning the variance between groups was unequal, Welch’s *F* has been reported with Games-Howell post hoc test results. When variance between groups was equal, as determined by the Levene’s test, Hochberg’s GT2 post hoc tests were used. For the ANOVA results, effect size (*r*) was calculated by calculating eta squared, then deriving the square root of this number (Field 2013). Recommended cut-offs for *r* are > .10 = small effect, > .30 = medium effect and > .50 = large effect. Thus, effect sizes < .10 were not deemed of interest, so post hoc results are not reported. For post hoc tests, Cohen’s *d* effect sizes were calculated.

For the independent samples *t* tests investigating gender effects and the one-way ANOVAs investigating the effect of marital status, *p* values are reported uncorrected for repeated measures. However, as there are 6 TEAQ subscales, a Bonferroni correction was applied and only *p* values < .0083 deemed significant.

Pearson’s correlation coefficients (*r*) were calculated to investigate the relationship of each TEAQ subscale with age. Due to the sample size, very small correlation coefficients were significant, so only those greater than .3, explaining at least 9% of the variance were deemed of importance. Due to Hall and Veccia (1990) identifying an influence of gender on the relationship between age and touch behaviours, correlations were additionally determined for each gender separately. For all analyses, normality was assumed due to the central limit theorem.

### Results

#### The Effect of Gender on TEAQ Responses

**Fig. 1:**
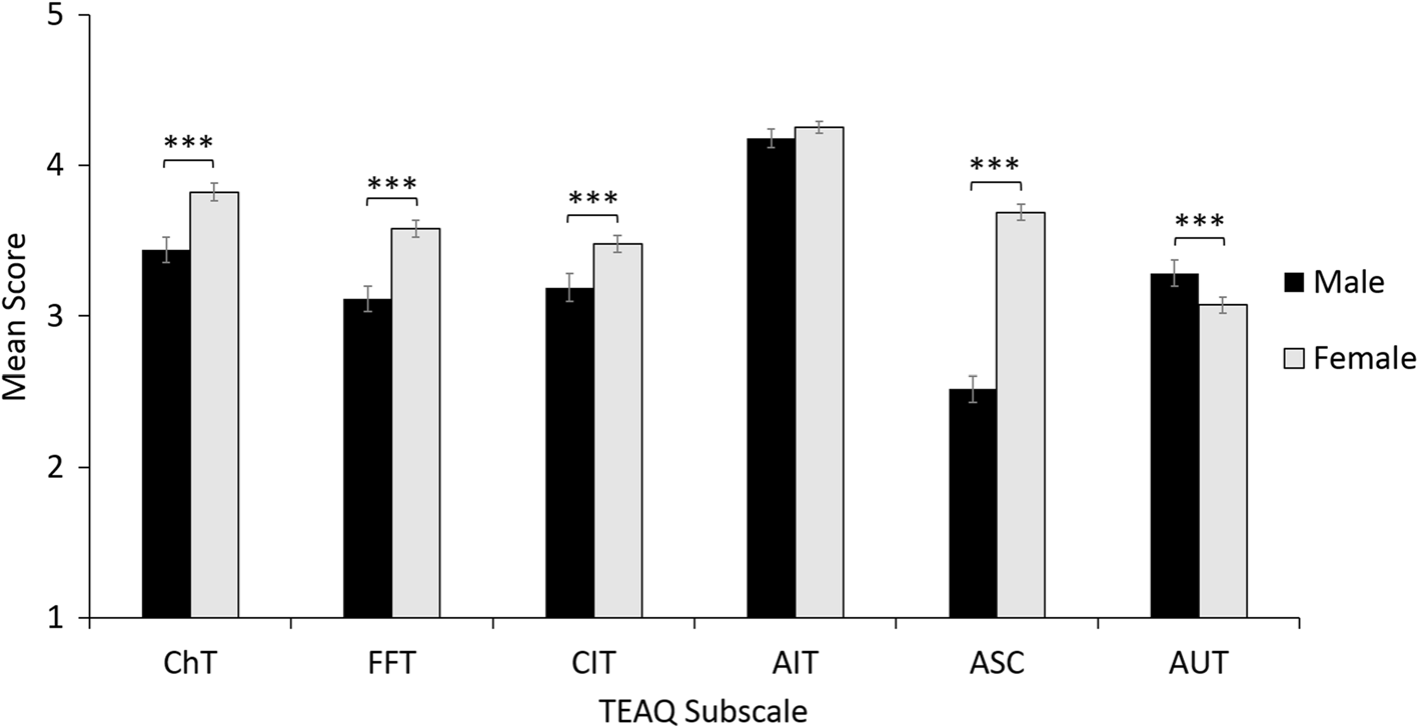
Gender differences in TEAQ subscale scores. Mean scores with 95% CI error bars are shown. *TEAQ* Touch Experiences and Attitudes Questionnaire, *ChT* Childhood Touch, *FFT* Friends and Family Touch, *CIT* Current Intimate Touch, *AIT* Attitude to Intimate Touch, *ASC* Attitude to Self-Care, *AUT* Attitude to Unfamiliar Touch. Significant differences are indicated, ***p < .001

The participant reporting their gender as ‘other’ was not included in the analysis, as a single response did not enable any group comparisons and so the analysis compared responses of males to females. The sample for the analysis of gender effects consisted of 1106 females and 401 males. As shown in Fig. 1, independent samples *t* tests revealed the TEAQ FFT score for females (*M* = 3.58, *SD* = 0.96) was significantly greater than males (*M* = 3.11, *SD* = 0.88), with a medium effect size (*t*_768.748_ = 8.89, *p* < .001, Cohen’s *d* = 0.50). TEAQ CIT scores were significantly higher for females (*M* = 3.48, *SD* = 0.95) than males (*M* = 3.19, *SD* = 0.97), with a small effect size (*t*_1505_ = 5.19, *p* < .001, Cohen’s *d* = 0.30). TEAQ ChT scores were significantly higher for females (*M* = 3.82, *SD* = 0.99) than males (*M* = 3.44, *SD* = 0.85) with a small effect size (*t*_810.827_ = 7.38, *p* < .001, Cohen’s *d* = 0.40). TEAQ ASC scores were significantly higher for females (*M* = 3.69, *SD* = 0.90) than males (*M *= 2.52, *SD* = 0.89) with a large effect size (*t*_1505_ = 22.45, *p* < .001, Cohen’s *d* = 1.31). TEAQ AIT scores for males (*M* = 4.18, *SD* = 0.64) and females (*M* = 4.25, *SD* = 0.70) were comparable (*t*_770.458_ = 1.90, *p *= .057, Cohen’s *d* = 0.11). Finally, TEAQ AUT scores were significantly greater for males (*M* = 3.29, *SD* = 0.89) than females (*M* = 3.07, *SD* = 0.90) with a small effect size (*t*_1505_ = 4.06, *p* < .001, Cohen’s *d* = 0.24).

#### One-Way ANOVA for Effect of Marital Status

One-way ANOVAs were used to determine if there was any effect of marital status on TEAQ score. Total sample size was 1472 for this analysis (Males = 395, Females = 1075, ‘Other’ = 1, not stated = 1). There were 568 single participants, 528 participants in a relationship and 376 married/cohabiting participants.

Following Bonferroni correction (uncorrected *p* values are reported), no significant effect of marital status on TEAQ FFT (*F*_2,1469_ = 3.96, *p* = .019, *r* = 0.07) or TEAQ AUT was identified (*F*_2,1469_ = 0.95, *p* = .389, *r* = 0.04). A significant effect of marital status on TEAQ ChT was identified, just surviving correction for multiple comparisons, however, the effect size was not substantial (*F*_2,882.351_ = 4.83, *p* = .008, *r* = 0.08). A significant effect of marital status on TEAQ ASC was also identified and survived correction for multiple comparisons, however the effect size was not substantial (*F*_2,1469_ = 5.53, *p* = .004, *r* = 0.09).

**Fig. 2:**
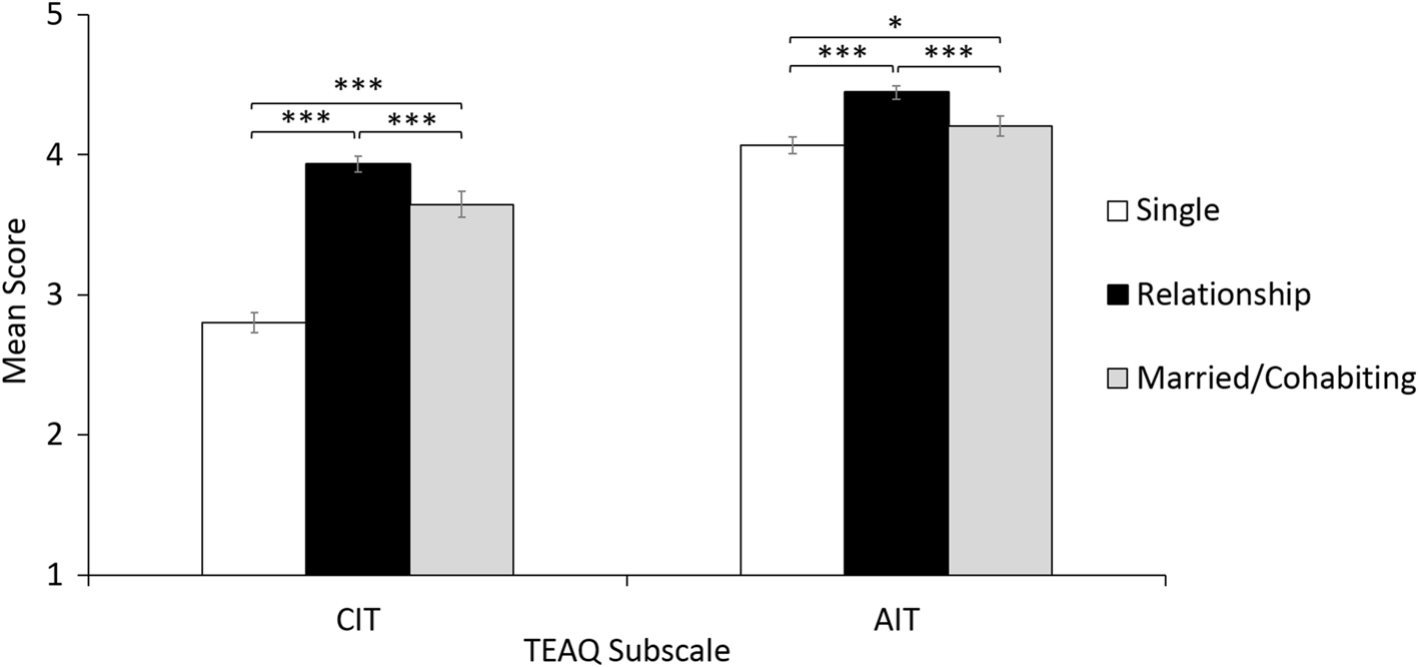
The effect of marital status on TEAQ subscale scores. Mean scores with 95% CI error bars are shown. *TEAQ* Touch Experiences and Attitudes Questionnaire, *CIT* Current Intimate Touch, *AIT* Attitude to Intimate Touch. Significant differences are indicated, ***p < .001; *p < .05

As shown in Fig. 2, a significant, large effect of TEAQ CIT was identified, (*F*_2,874.198_ = 302.60, *p* < .001, *r* = 0.53). Games-Howell post hoc analysis revealed all groups to be significantly different. TEAQ CIT score was significantly higher for those in a relationship (*M* = 3.93, *SD* = 0.67) compared to those who were single (*M* = 2.80, *SD* = 0.86) with a large effect size (*t*_1058.592_ = 23.24, *p* < .001, Cohen’s *d* = 1.46) and those married/cohabiting (*M* = 3.65, *SD* = 0.89) with a small effect size (*t*_657.638_ = 5.28, *p* < .001, Cohen’s *d* = 0.37). Those who were married/cohabiting had significantly greater TEAQ CIT scores than those who were single with a large effect size (*t*_784.212_ = 14.44, *p* < .001, Cohen’s *d* = 0.97).

A small, but significant effect of marital status on TEAQ AIT was identified and survived correction for multiple comparisons (*F*_2,877.854_ = 50.61, *p* < .001, *r* = 0.24). Games-Howell post hoc analysis revealed a significant difference between all groups. Those in a relationship had a significantly more positive attitude to intimate touch (*M* = 4.44, *SD* = 0.54) than those who were single (*M* = 4.07, *SD* = 0.73) with a medium effect size (*t*_1040.038_ = 9.75, *p* < .001, Cohen’s *d* = 0.58) and those who were married/cohabiting (*M* = 4.20, *SD* = 0.71) with a small effect size (*t*_664.776_ = 5.53, *p* < .001, Cohen’s *d* = 0.39). Those who were married/cohabiting had a significantly greater TEAQ AIT score than those who were single with a non-substantial effect size (*t*_818.978_ = 2.84, *p* = .013, Cohen’s *d* = 0.19).

#### Age

Eight participants did not report their age, so were excluded from the analysis, leaving a sample size of 1501 (1100 females, 398 males, 1 ‘Other’, 2 not stated). Pearson’s *r* for all factors was < .3, so no correlations of interest were identified (TEAQ FFT: *r* = − .03, *p* = .211; TEAQ CIT: *r* = − .13, *p* < .001; TEAQ ChT: *r* = − .21, *p* < .001; TEAQ ASC: *r* = − .09, *p* < .001; TEAQ AIT: *r* = − .09, *p* < .001; TEAQ AUT: *r* = − .004, *p* = .877). Analysis of females only still identified no correlation coefficients > .3 (TEAQ FFT: *r* < .001, *p* = .995; TEAQ CIT: *r* = − .14, *p* < .001; TEAQ ChT: *r* = − .21, *p* < .001; TEAQ ASC: *r* = − .09, *p* = .003; TEAQ AIT: *r* = − .11, *p* < .001; TEAQ AUT: *r* = .02, *p* = .521). Similarly, for males only, no correlation coefficients > .3 were identified (TEAQ FFT: *r* = − .09, *p* = .062; TEAQ CIT: *r* = − .10, *p* = .050; TEAQ ChT: *r* = − .19, *p* < .001; TEAQ ASC: *r* = − .09, *p* = .088; TEAQ AIT: *r* = − .06, *p* = .253; TEAQ AUT: *r* = − .08, *p* = .102).

### Discussion

As predicted, gender differences in TEAQ responses were identified, with females having greater FFT, CIT, and ChT scores than males, suggesting that overall, females appear to experience more physical touch throughout their lifetimes than males. This is supported by previous literature which has identified females to experience more positive touch both during childhood (Lindahl and Heimann 1997; Takeuchi et al. 2010) and in adulthood (Hall and Veccia 1990; Jones 1986; Major et al. 1990; Webb and Peck 2015). As predicted, females had a more positive attitude to self-care than males, a result support by previous literature that females have significantly greater self-reported attitudes to body care than males in adolescence (Brausch and Muehlenkamp 2007). In adulthood, use of personal care products and investment in appearance has been identified as greater in females than males (Biesterbos et al. 2013; Muth and Cash 1997).

Interestingly, attitude to intimate touch was comparable between males and females, so the greater amount of intimate touch reported by females does not appear to be driven by a stronger desire for intimate touch. Additionally, males had a more positive attitude to unfamiliar touch. The prediction that females would have a more positive attitude to touch was therefore only supported for self-care, but not for intimate or unfamiliar touch. Greater touch avoidance, particularly relating to the opposite-sex, has been previously identified in females compared to males (Andersen et al. 1987; Guerrero and Andersen 1994), supporting this result and suggesting this difference in touch avoidance is context dependent, with higher levels in females particularly for touch with unfamiliar individuals.

As expected based on previous literature (Guerrero and Andersen 1991), individuals in a romantic relationship reported significantly greater amounts of current intimate touch than those married/cohabitating, which in turn was greater than the current intimate touch reported for single participants. The same pattern of results was identified for the attitude to intimate touch scale, although the effect size was smaller. These results further support the construct validity of the TEAQ CIT and AIT scales.

No effect of age on TEAQ responses was identified, reflecting a complicated relationship between age and physical touch identified in the literature. The finding of no overall effect of age on touching behaviour is supported by those of Hall and Veccia (1990). It should be considered that participant recruitment for the current study was not stratified by age. As such 74% of participants were less than 30 years old, which may in part explain why no significant effects of age were identified. A more thorough examination of whether TEAQ responses alter throughout adulthood is required. Although most participants were less than 30, the oldest participant was 81, so the fact responses did not change with age suggests the TEAQ may well be suitable for use with adults of all ages.

In conclusion, the known-group validity of the TEAQ is good with expected group differences identified and supported by the literature.

## General Discussion

This article describes the construction and validation of the Touch Experiences and Attitudes Questionnaire (TEAQ), a self-report measure of an individual’s experiences of positive touch both in childhood and at present and their current attitudes to positive touch, in terms of interpersonal touch and self-care. The original TEAQ contained 117 items, encompassing all key circumstances in which positive touch occurs. This holistic approach was designed to allow an accurate measure of positive touch experiences and attitudes, without the influence of bias based on authors’ preconceptions.

PCA was carried out on data from the 117-item draft TEAQ. This identified a 57 item, 6-component structure. Components related to positive touch experiences in childhood (ChT), touch between friends and family (FFT), current experience of intimate touch (CIT), attitude to intimate touch (AIT), attitude to touch with unfamiliar people (AUT) and attitude to self-care (ASC). Components had high Cronbach’s α, suggesting good component reliability. The reliability of the 6-component structure was confirmed using CFA on a second dataset of responses to the shortened, 57-item TEAQ. The face validity of the TEAQ has been identified to be good, with subscale names reflecting subscale items well.

The importance of positive touch in childhood is widely accepted and it is recognized that positive touch has a key role in early development. It is widely accepted that positive touch experiences in the early developmental period have a key role in the healthy development of a child (Bowlby 1951; Harlow 1958; Harlow and Suomi 1970; Spitz 1945), with epigenetic mechanisms implicated (Meaney and Szyf 2005; Murgatroyd et al. 2015). As such, it is to be expected that positive touch in childhood was identified as a factor of the TEAQ.

Predictive and discriminant validity of the TEAQ ChT subscale was identified in Study 3. The TEAQ ChT subscale was significantly negatively predictive of childhood trauma, explaining significantly more of the variance in childhood trauma than either the FTO or QPCE childhood subscale scores alone. Additionally, when investigating which childhood trauma subscales were predictive of the TEAQ ChT subscale, childhood emotional neglect was the only significant subscale. This is of interest as we know positive touch, particularly stroking touch, is important in the communication of love (App et al. 2011; Hertenstein et al. 2009) and that stroking touch activates C-tactile afferents, implicated in the encoding of affective rather than discriminatory touch (see McGlone et al. 2014 for a review). That positive touch in childhood is most strongly negatively related to childhood emotional neglect, highlights a key emotional component of positive interpersonal touch in childhood.

Current experiences of positive touch have been identified to promote well-being and be protective against depression (Cochrane 1990; Uvnäs-Moberg et al. 2015). Additionally, positive touch in terms of massage therapy has therapeutic benefits; reducing depression, stress, anxiety, aggression, and pain (Diego et al. 2002; Field et al. 1996, 2004; Hernandez-Reif et al. 1998, 2001; Liljencrantz et al. 2017; Liljencrantz and Olausson 2014). Touch responses have been identified to be context dependent; even when the same touch is delivered, significant differences in central responses have been identified by manipulating the context in which the touch occurs (Gazzola et al. 2012; McCabe et al. 2008). It is therefore not surprising that both attitudes to intimate touch (AIT) and current experiences of intimate touch (CIT) were identified as distinct factors from the context in which touch occurs between friends and family (FFT), with unfamiliar people (AUT) and in terms of self-care (ASC).

It is of interest that the CIT subscale, rather than the FFT subscale was significantly predictive of satisfaction with social support. It has been previously identified that love and sympathy are emotions which individuals prefer to communicate non-verbally via touch rather than with facial expressions or body posture (App et al. 2011). The CIT rather than FFT contains items relating to receiving sympathy in terms of consoling touch, as well as receiving stroking touch, identified as involved in the communication of love (Hertenstein et al. 2009). Key components of positive touch in relation to social support can thus be related more strongly to the CIT subscale compared to the FFT subscale.

Of the TEAQ subscales, the subscale relating to attitudes to touch with unfamiliar people (AUT) was most strongly negatively associated with the Wilhelm et al. (2001) STQ. The STQ was developed for a study investigating social anxiety, with highly socially anxious participants scoring significantly higher on the STQ than those with lower social anxiety. Further investigation of whether AUT scores relate to social anxiety would be of interest.

The attitude to self-care (ASC) subscale was the only factor identified which related to non-interpersonal touch. These items can be related to those of Orbach and Mikulincer’s (1998) Body Care subscale of the Body Investment Scale, which contains items relating to taking a bath, using body care products, and pampering the body. Orbach and Mikulincer (1998) identified suicidal adolescent inpatients had significantly lower body care scores than healthy controls. Additionally, it has been identified that those with severe mental health difficulties may neglect their physical appearance and personal hygiene, reflecting a neglect in self-care (Corrigan 2000; First et al. 2002; Häfner et al. 2003). Investigating TEAQ ASC subscale scores in relation to psychopathology, particularly depression would therefore be of interest.

As described in Study 4, known-group validity was identified to be good for the TEAQ subscales, with significant differences in terms of gender and marital status identified. Gender differences were most pronounced for the ASC subscale, reflecting a more positive attitude to self-care in females than males. This result is supported by previous literature that females have significantly greater self-reported attitudes to body care, greater use of personal care products and invest in their appearance more than males (Biesterbos et al. 2013; Brausch and Muehlenkamp 2007; Muth and Cash 1997).

It is of interest that items relating to non-interpersonal touch in terms of touching fabrics or animals were not included in the final TEAQ and were therefore identified as not substantially related to the underlying construct measured by the TEAQ. In terms of touch with animals, this is supported by the result identified in Study 3, that the item relating to touch with animals in Wilhelm et al.’s (2001) STQ had an extremely low correlation with total STQ score, suggesting this item not to be related to the underlying construct the STQ is measuring. Although touch with animals has been identified as rewarding and beneficial in terms of promoting well-being (Odendaal and Meintjes 2003; Uvnäs-Moberg et al. 2015), interpersonal touch, rather than touch with animals appears to be of particular relevance.

It is not necessarily surprising that questions relating to touching fabrics were not strongly related to the underlying construct of the TEAQ, however, this should be noted considering a large amount of research in the field of affective touch involves participants responding to robotic touch (e.g., Ackerley et al. 2014; Essick et al. 2010; Loken et al. 2009) and touch delivered with soft brushes (e.g. Bjornsdotter et al. 2009; Kaiser et al. 2015; Olausson et al. 2002, 2008; Trotter et al. 2016). It has to be considered that although these touches are pleasant and positive and have the advantage of being well-controlled, their ecological validity is relatively low and may not be as strongly related to physical touch responses in a real-world setting as we would like to believe. Combining these well-controlled touches with a self-report measure such as the TEAQ can help improve these studies, providing quality data from a laboratory setting with self-report data about an individual’s typical physical touch attitudes and behaviors in their everyday life. Although numerous observational studies about tactile behaviors have been conducted (e.g., Hall and Veccia 1990; Major et al. 1990; Remland et al. 1995), these are obviously limited by touch occurring in public places and cannot access individual’s attitudes towards touch, which is why a well validated self-report measure of touch experiences and attitudes, such as the TEAQ, will be of value for inclusion in physical touch research studies.

In terms of the limitations of this study, participants were predominantly recruited through university settings, therefore further investigation is required to determine the validity of the TEAQ for use with in-patients and other non-community-based samples. A further consideration is most participants did not have children. It is reasonable to suggest parenthood may alter touch experiences and attitudes, so further investigation of a sample including more parents could be of value. For Study 3, the number of participants who had experienced childhood trauma in the sample was low. Examination of total CTQ scores, identified the modal score (representing 13.4% of participants) was the minimum score possible. Repetition of this study using a sample of participants with higher levels of childhood adversity, such as care leavers, would be beneficial.

Individual differences in tactile sensitivity have been documented (e.g. Magerl et al. 2010; Rolke et al. 2006), but were not considered in this investigation. In addition, more generalized individual differences in sensory-processing sensitivity have been identified, with Aron and Aron (1997) developing the Highly Sensitive Person Scale to identify individuals with high sensory-processing sensitivity. Investigating how individual differences in sensory-processing sensitivity influence touch experiences and attitudes as measured by the TEAQ would be of particular interest.

Cultural norms in terms of physical touch behaviours vary (Field 1999; Jourard 1966; Remland et al. 1995). The majority of participants in these studies were white British, so further validation in other cultures is required. Although the age range for these studies was reasonable, the majority were less than 30 years old, so validation of this questionnaire for use with older adults is also required.

Additionally, this measure purposefully avoided making the distinction between same- versus opposite-gender touch. This was to allow touch attitudes and experiences to be determined without any confounds relating to sexuality or attitudes towards homosexuality. However, it is important to consider the distinction between same- versus opposite-gender touch. For instance, higher levels of same-gender touch avoidance have been reported for males than females (Andersen and Leibowitz 1978). Additionally, an observational study by Hall and Veccia (1990) identified same-gender touches to be more frequent between females than males, whereas initiation of opposite-gender touches was relatively similar for males and females, although this varied by age. The TEAQ could be adapted for future investigations to allow differentiation between same-versus opposite-gender touch.

The validity of using the subscales as independent questionnaires has yet to be determined, but as Cronbach’s alpha has been demonstrated to be high for all subscales, it is likely this would yield reliable results and would be a reasonable use of the TEAQ. The high predictive validity of the childhood subscale in particular has been demonstrated, suggesting this subscale may be particularly useful as a stand-alone measure.

In conclusion, this study has demonstrated the TEAQ to have good face validity, internal consistency, construct validity in terms of discriminant validity, known-group validity, and convergent validity, and criterion-related validity in terms of predictive validity and concurrent validity. To the best of the authors’ knowledge, the TEAQ is the only physical touch questionnaire currently available which provides a measure of both touch experiences and attitudes and for which the factor structure has been determined and validated. We anticipate this questionnaire will be a valuable tool for the field of physical touch research.

### Electronic supplementary material

Below is the link to the electronic supplementary material.

Supplementary material 1 (DOCX 390 kb)

## Appendix 1: Touch Experiences and Attitudes Questionnaire (TEAQ)

Please select a response next to each of the statements below to indicate how much you agree or disagree with each statement

**Table:**

	Disagree strongly	Disagree a little	Neither agree nor disagree	Agree a little	Agree strongly
1. I dislike people being very physically affectionate towards me					
2. I like using body lotions					
3. I have to know someone quite well to enjoy a hug from them					
4. I find it natural to greet my friends and family with a kiss on the cheek					
5. There was a lot of physical affection during my childhood					
6. As a child I would often hug family members					
7. I like to use bath essence when having a bath					
8. I find stroking the hair of a person I am fond of very pleasurable					
9. My parents were not very physically affectionate towards me during my childhood					
10. I like to fall asleep in the arms of someone I am close to					
11. I often snuggle up on the sofa with someone					
12. I enjoy the physical intimacy of sexual foreplay					
13. I like to link arms with my friends and family as I walk along					
14. I usually hug my family and friends when I am saying goodbye					
15. As a child I found a hug from my parents when I was upset made me feel much happier					
16. It’s nice when friends and family members greet me with a kiss					
17. I often hold hands with someone I know intimately					
18. When I am upset, there is usually someone who can comfort me.					
19. Kissing is a great way of expressing physical attraction					
20. It feels really good when someone I am fond of runs their fingers through my hair					
21. I regularly hug people I am close to					
22. As a child my parents would tuck me up in bed every night and give me a hug and a kiss goodnight					
23. My life lacks physical affection					
24. I enjoy having my skin stroked					
25. I often take a shower or bath with someone					
26. I enjoy having sex					
27. I often have sex					
28. I am put off by physical familiarity					
29. I can always find somebody to physically comfort me when I am upset					
30. I always greet my friends and family by giving them a hug					
31. I enjoy being cuddled by someone I am fond of					
32. My mother regularly bathed me as a child					
33. As a child my parents always comforted me when I was upset					
34. I enjoy the feeling of my skin against someone else’s if I know them intimately					
35. As a child my parents would often hold my hand when I was walking along with them					
36. Most days I get a hug or a kiss					
37. If someone I don’t know very well puts a friendly hand on my arm it makes me feel uncomfortable					
38. I often make physical contact with my friends and family when I am with them					
39. It makes me feel uncomfortable if someone I don’t know very well touches me in a friendly manner					
40. I enjoy holding hands with someone I am fond of					
41. I often share a romantic kiss					
42. As a child my mother regularly brushed my hair					
43. I like exfoliating my skin					
44. Kissing is an enjoyable part of expressing romantic feeling					
45. I often have my skin stroked					
46. I often hold hands with someone I am fond of					
47. I like to stroke the skin of someone I know intimately					
48. I am on huggable terms with quite a few people					
49. I often fall asleep while holding someone I am close to					
50. Snuggling up on the sofa with someone is great					
51. I often put my arm around a close friend as we walk along together					
52. I like having a bath with lots of bubble bath					
53. I don’t get many hugs these days					
54. I am often given a shoulder massage					
55. I like to use face masks on my skin					
56. I like it when my friends and family greet me by giving me a hug					
57. I often link arms with my friends and family as I walk along					

Scoring: Disagree strongly = 1, disagree a little = 2, neither agree nor disagree = 3, agree a little = 4, agree strongly = 5

**R** denotes items which are reverse scored (i.e. disagree strongly = 5, disagree a little = 4, neither agree nor disagree = 3, agree a little = 2, agree strongly = 1). Item numbers below indicate the items which belong to each of the subscales

Calculate the mean score for each subscale to obtain a subscale score

*Friends and family touch (FFT)* (*11 items*): 4, 13, 14, 16, 21, 30, 38, 48, 51, 56, 57

*Current intimate touch (CIT)* (*14 items*): 11, 17, 18, 23**R**, 25, 27, 29, 36, 41, 45, 46, 49, 53**R**, 54

*Childhood touch* (*ChT*) (*9 items*): 5, 6, 9**R**, 15, 22, 32, 33, 35, 42

*Attitude to self*-*care* (*ASC*) (*5 items*): 2, 7, 43, 52, 55

*Attitude to intimate touch* (*AIT*) (*13 items*): 8, 10, 12, 19, 20, 24, 26, 31, 34, 40, 44, 47, 50

*Attitude to unfamiliar touch* (*AUT*) (*5 items*): 1**R,** 3**R**, 28**R**, 37**R**, 39**R**
