# Supplementary material for: Construction and Validation of the Touch Experiences and Attitudes Questionnaire (TEAQ): A Self-report Measure to Determine Attitudes Toward and Experiences of Positive Touch
Source: J Nonverbal Behav. 2018 Aug 1;42(4):379–416. doi: 10.1007/s10919-018-0281-8 (PMC6208655; doi:10.1007/s10919-018-0281-8)
Supplement: Supplementary file 1 — Supplementary material 1 (DOCX 390 kb) [file 10919_2018_281_MOESM1_ESM.docx]

**Online Supplementary Materials**

**Original TEAQ CFA model**

**Fig. S1** Path Diagram of the original component structure of the Touch Experiences and Attitudes Questionnaire (TEAQ) for Confirmatory Factor Analysis (CFA). Abbreviations: FFT – Friends and Family Touch, CIT – Current Intimate Touch, ChT – Childhood Touch, ASC – Attitude to Self-Care, AIT – Attitude to Intimate Touch, AUT – Attitude to Unfamiliar Touch.

**Parcelled TEAQ CFA model**

**Fig. S2** Path Diagram of the parcelled component structure of the Touch Experiences and Attitudes Questionnaire (TEAQ) for Confirmatory Factor Analysis (CFA). Abbreviations: FFT – Friends and Family Touch, CIT – Current Intimate Touch, ChT – Childhood Touch, ASC – Attitude to Self-Care, AIT – Attitude to Intimate Touch, AUT – Attitude to Unfamiliar Touch.
